# Dynamic Emotion Recognition and Expression Imitation in Neurotypical Adults and Their Associations with Autistic Traits

**DOI:** 10.3390/s24248133

**Published:** 2024-12-19

**Authors:** Hai-Ting Wang, Jia-Ling Lyu, Sarina Hui-Lin Chien

**Affiliations:** 1 Graduate Institute of Biological Science and Technology, China Medical University, Taichung 404, Taiwan; drwanghaiting88@gmail.com; 2 Graduate Institute of Biomedical Sciences, China Medical University, Taichung 404, Taiwan; u105306601@cmu.edu.tw; 3 Neuroscience and Brain Disease Center, China Medical University, Taichung 404, Taiwan

**Keywords:** face processing, dynamic emotion recognition, expression imitation, autism quotient (AQ), broader autism phenotype (BAP)

## Abstract

Autism spectrum disorder (ASD) is a neurodevelopmental disorder characterized by deficits in social interaction and communication. While many studies suggest that individuals with ASD struggle with emotion processing, the association between emotion processing and autistic traits in non-clinical populations is still unclear. We examine whether neurotypical adults’ facial emotion recognition and expression imitation are associated with autistic traits. We recruited 32 neurotypical adults; each received two computerized tasks, the Dynamic Emotion Recognition and Expression Imitation, and two standardized measures: the Chinese version AQ and the Twenty-Item Prosopagnosia Index (PI-20). Results for the dynamic emotion recognition showed that happiness has the highest mean accuracy, followed by surprise, sadness, anger, fear, and disgust. For expression imitation, it was easiest to imitate surprise and happiness, followed by disgust, while the accuracy of imitating sadness, anger, and fear was much lower. Importantly, individual AQ scores negatively correlated with emotion recognition accuracy and positively correlated with PI-20. The AQ imagination, communication sub-scores, and PI-20 positively correlated with the expression imitation of surprise. In summary, we found a significant link between recognizing emotional expressions and the level of autistic traits in non-clinical populations, supporting the concept of broader autism phenotype.

## 1. Introduction

Autism spectrum disorder (ASD) is a neurodevelopmental disorder characterized by deficits in social interaction and communication and by the presence of restricted and stereotyped behaviors and sensory anomalies (DSM-5, American Psychiatric Association, 2013) [1]. Deficits in processing affect seem to be intrinsic to individuals with ASD, which plays an important role in their social dysfunction [2]. Human faces provide a wealth of information essential in daily life, not only for identifying others’ gender, race, and age but also for understanding their intentions, thoughts, and emotions. Many studies have confirmed that an impairment in face processing in ASD is widespread [3,4,5] and present from an early age [6,7]. This impairment in face processing also affects the ability to identify and interpret emotions from facial expressions [8,9], which may contribute to difficulties in social and communicative development commonly observed in ASD [10,11].

In addition to recognizing emotions, appropriate facial expressions play a crucial role in reciprocal social communication. Research has shown that producing elicited affective expressions is more difficult for individuals with autism than individuals with Down’s syndrome of similar chronological age, mental age, and IQ [12]. A recent study utilizing computer-based facial analysis demonstrated that autistic individuals imitated facial expressions more slowly and with less precision than neurotypical individuals across different emotions [13]. Facial EMG evidence suggested that participants with ASD had delayed spontaneous mimicry for various emotional expressions, including happy, sad, fearful, angry, disgusted, and neutral expressions [14]. In studies where facial mimicry was videotaped in response to dynamic expressions of anger and happiness, a reduced response was notably observed in individuals with high-functioning ASD [15,16]. Qualitatively, autistic participants exhibited less differentiated, awkward, or “mechanical” expressions [17]. These overt impairments in expression mimicry or production may underlie difficulties in social interaction among individuals with ASD.

Human infants show a natural preference for faces [18,19,20] and are sensitive to basic facial emotions [21,22]. However, their ability to recognize and understand emotional expressions improves significantly from preschool through middle childhood and adolescence [23,24,25,26,27]. Facial expressions are essential visual cues that help convey and share intentions and emotional states. The “theory of universal emotions” suggests that individuals from various cultures can identify at least six basic facial expressions (i.e., happiness, sadness, anger, fear, surprise, and disgust) [28,29]. When children were asked to match static photographs or cartoon characters with one of six basic emotions, their expression perception improved between the ages of 6 and 8, with a further improvement to adult performance around age 14 [27]. Using the static Ekman–Friesen Pictures of Facial Affect, researchers studied 6- to 16-year-old children and found that happiness was most accurately recognized; 6-year-olds recognized sadness and anger at levels comparable to that of 16-year-olds. However, disgust, fear, and surprise were identified as the “later-developing” emotions that continue to improve in late childhood [30].

Emotional expressions in everyday interactions are diverse in both motion and intensity. Using dynamic stimuli, such as morphing techniques (which involve creating a series of images changing from a neutral state to a more intensely expressed emotion), may enhance our ability to recognize subtle differences and variations in how these expressions are interpreted [31,32,33]. Children and adolescents were less sensitive to sadness, fear, and anger than adults when using the dynamic Ekman facial expressions [31,32]. Richoz et al. studied how people recognize static and dynamic facial expressions across lifespans. They discovered that overall recognition accuracy is generally higher for dynamic expressions. Five-year-old children could recognize “happiness” as effectively as adults; they also recognized anger and sadness well at an early age. However, a gradual improvement was observed, primarily in recognizing static expressions. Consistent with previous studies using static stimuli, the recognition of surprise, fear, and sadness developed later, showing a steeper increase with the static stimuli [26].

Facial expression is another important social skill that emerges early in life. Neonates can imitate facial gestures, such as tongue protrusion and mouth opening [34,35]. This ability to imitate enables infants to quickly establish reciprocal relationships with their caregivers, which enhances their learning and chances of survival [36]. Three to five-year-old preschool children had superior skills in producing facial expressions than discriminating them. They were more accurate at expressing happiness than sadness, surprise, disgust, fear, or anger during imitation tasks [37]. Children continued learning to generate or imitate facial expressions, and overall accuracy increased from ages 6 to 11. Among the six basic emotions, joy (or happiness) was more accurately produced than anger and sadness [38].

Many studies explored the relationship between emotion recognition and facial expression imitation. Neurotypical adults tend to generate facial expressions that match social interaction automatically. For example, in one of the early studies, Dimberg found that participants showed more Corrugator Supercilii (brow) activity when exposed to anger expression and more Zygomaticus Major (cheek) activity when exposed to happy expression separately [39]. Facial mimicry may reduce perceived emotion recognition task difficulty because it provides additional motor information regarding the expressions to decode [40]. Facial expression imitation may be part of emotion recognition [41]. Blocking facial mimicry impaired recognition of emotional expressions differentially among happiness, disgust, fear, and sadness, with the recognition of happiness most severely impaired [42]. The ability to judge the genuineness of true or false smiles dropped if facial mimicry was inhibited [43]. Through sensorimotor networks, simulating a perceived facial expression might help infer the underlying emotion of the interacting partner and contribute to facial expression recognition [44].

Autistic traits are proposed to be continuously distributed in the general population. This perspective suggests that individuals exhibiting the non-clinical or sub-clinical broader autistic phenotype (BAP) display mild yet qualitatively comparable characteristics to those diagnosed with ASD [45]. Furthermore, neurotypical adults who possess elevated levels of autistic traits often face significant difficulties in emotional expression recognition, highlighting the pervasive impact of these traits beyond clinical diagnoses. Poljac et al. used a dynamic emotion recognition task and demonstrated a reduced accuracy and sensitivity for the emotions of anger, disgust, and sadness in the broader autistic phenotype [46]. In a study examining event-related potentials in non-clinical adolescents, the high-AQ group displayed significantly lower late positive potentials (LPPs) during emotional processing [47]. Emotion recognition accuracy in adults with Anorexia Nervosa was negatively correlated with autistic features, meaning that the higher the autistic trait, the worse the recognition performance [48]. It has been reported that facial imitation improves emotion recognition in adults with different levels of sub-clinical autistic traits [49]. Kowallik et al. investigated the influence of expression imitation on facial emotion recognition. They found that emotion recognition and expression imitation were both improved by the instruction to imitate. Importantly, people with higher sub-clinical autistic traits exhibited a significantly greater improvement in emotion recognition.

The relationship among autistic traits, emotion recognition, and expression imitation remains unclear and requires further exploration. Enhancing emotion processing may help improve social interactions. Therefore, exploring the connection between emotion recognition and facial expression imitation could provide potential interventions to improve social interaction skills in individuals with clinical and sub-clinical ASD. Our main objective is to examine the relationships between autistic traits, emotion recognition, and facial expression imitation in neurotypical adults. Specifically, we aim to determine how an individual’s ability to process emotion (including perception and production) correlates with autistic traits in the non-clinical population. To tackle these questions, we used a within-subject design in which each participant received an AQ questionnaire (to assess autistic traits), a Twenty Item Prosopagnosia Index (PI20) questionnaire (a subjective evaluation of problems with face recognition in daily life), and two computerized tasks. One was the *Dynamic Facial Emotion Recognition Task*, in which we asked the participants to recognize six basic emotions (Happy, Anger, Sadness, Surprise, Fear, and Disgust) in clips of facial emotions transforming from 0% (neutral) to 100% (full-intensity) state. The other one was the *Expression Imitation Task*. We instructed participants to watch a short clip displaying the Chinese labels and facial images of the six basic emotions and imitate them. Simultaneously, their facial imitations were recorded. We used the iMotions^TM^ Affectiva software 7.1 for emotional expression coding, categorizing, and digitalizing imitation accuracy. This software allowed us to analyze the participants’ responses during the expression imitation tasks, enabling a comprehensive assessment of the correlation between their performance and autistic traits.

## 2. Materials and Methods

### 2.1. Participants

We recruited 32 neurotypical adults aged between 19 and 32 with no self-reported history of psychiatric or neurological disorders (n = 32, mean age = 22.9 years, *SD* = 3.2; 16 females, mean age = 22.6, *SD* = 3.7; 16 males, mean age = 23.2, *SD* = 2.7). This sample size was calculated according to Faul et al. [50] and was determined based on G*Power calculation (i.e., we set the type 1 error (α) to 0.05, the power (*1-β*) to approximately 0.80 and medium effect size, leading to an estimated sample size of 34). Most participants were undergraduate or graduate students from China Medical University, Taichung, Taiwan. Each participant had normal or corrected-to-normal vision (20/20) and joined the study voluntarily. Informed written consent was obtained from each participant before the study. The present study adhered to the ethics of scientific publication as detailed in the *Ethical Principles of Psychologists and the Code of Conduct* [51] and to the Committee on Publication Ethics (COPE) guidelines. The participants were informed that their facial expression data were being protected and their anonymity guaranteed. The Institution Review Board of the Research Ethics Committee approved the study protocol at China Medical University Hospital, Taichung, Taiwan (Certificate number: CMUH110-REC2-260). In one lab visit, each participant received (1) the Chinese version of the Autism Quotient (AQ) questionnaire, (2) the Dynamic Facial Emotion Recognition task, (3) the Emotion Imitation task, and (4) the Prosopagnosia Index Twenty Items (PI20) questionnaire. The total duration of the study was about 30 min. Each participant received cash compensation at the end of the study.

### 2.2. The Autism-Spectrum Quotient Assessment

The Autism-Spectrum Quotient (AQ) is a self-rated questionnaire developed to measure autistic traits in adults with normal intelligence. In the present study, participants evaluated themselves with the Chinese version of the Autism-Spectrum Quotient (Liu, 2008) [52], adopted from Baron-Cohen et al. (2001) [53]. The AQ questionnaire consists of 50 questions assessing five areas: social skill, attention switching, attention to detail, communication, and imagination, with 10 questions for each area. Participants responded on a 4-point scale by indicating whether they “definitely disagree”, “slightly disagree”, “definitely agree”, or “slightly agree” with each statement. For the 25 positive questions, every answer in slight or strong agreement with an autistic behavior adds one point to the total score. For the 25 reverse questions, every answer in slight or strong disagreement adds one point to the total score. The full score is 50; the higher the score, the higher the autistic-like trait. The AQ is valuable for quantifying to what extent an individual possesses autistic traits and identifying where an autistic tendency is on the continuum from normality to autism. The cutoff score is 30 for the Chinese version AQ (c.f., 32 for the English version).

### 2.3. The Prosopagnosia-Index 20 Items (PI20)

The Twenty Item Prosopagnosia Index (PI20) is a self-report questionnaire for assessing face recognition difficulties, particularly in people with developmental prosopagnosia (DP), a condition where individuals have lifelong difficulties recognizing faces despite having normal vision and cognitive abilities [54,55]. It consists of 20 statements describing real-life experiences in many aspects of face recognition, which were collected from qualitative and quantitative descriptions. Participants responded on a 5-point scale (strongly agree to strongly disagree) by indicating to what extent the statements describe their experience. For the 15 positive statements, “strongly agree” scored 5 points, and “strongly disagree” scored 1 point. For the five reverse questions, “strongly agree” scored 1 point, and “strongly disagree” scored 5. The total score ranges from 20 to 100. The higher the score, the more problems with faces. A score over 65 may be suggestive of developmental prosopagnosia [54]. To make the PI20 easier for the participants, we translated it into Traditional Chinese in the present study.

### 2.4. Dynamic Facial Emotion Recognition Task

#### 2.4.1. Apparatus and Stimuli

We used a 22″ monitor (Acer P221W, Acer Inc., Taipei, ROC) and a desktop computer (Lemel LX3-TKBJ19-4S50A, Synnex, Taipei, ROC) to run the task. We applied E-Prime 2.0 (Psychology Software Tools, Sharpsburg, PA, USA) to execute the experimental program and to record the participant’s responses. The stimuli were the six basic emotion expressions for one female face and one male face (from the Taiwan Facial Expression Image Database TFEID) [56]. By employing FantaMorph 5 Deuluxe (Abrosoft Co., Lincoln, NE, USA), we were able to create 12 GIF videos of facial emotion expressions consisting of a series of morphing faces starting from neutral to full intensity with the six basic emotions (happy, anger, sadness, surprise, fear and disgust −100% intensity) for each gender (please see Chien et al. [57], Chen et al. [58], and Chiang et al. [31] for the detailed procedures of creating the morphing face stimuli). Each dynamic facial emotion expression GIF video clip played in full color at 30 frames per second. The image size on the screen was about 13.5 cm high and 13.5 cm wide. Each clip was about 2000 milliseconds long. Each participant received 60 video clip trials (12 videos × 5 repetitions) in random order.

#### 2.4.2. Procedures

**Figure 1:**
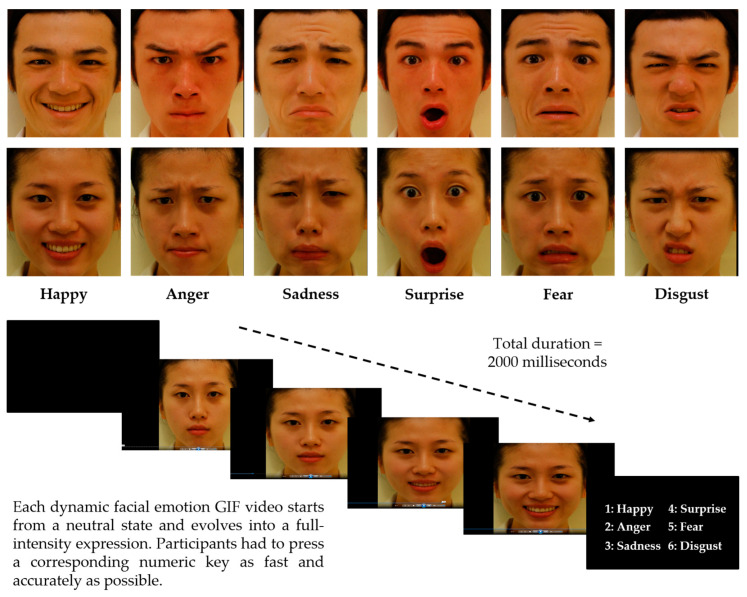
Diagram of the Dynamic Facial Emotion Recognition Task. The stimuli were the six basic emotion expressions (happy, anger, sadness, surprise, fear, disgust) for each gender (**top**). Each trial of the dynamic emotion recognition video starts at 0% and evolves to 100% intensity of the expression (**bottom**).

Each participant sat on a chair before the computer screen with a viewing distance of about 57 cm and adjusted their height to keep their eyes fixated on the screen center. Figure 1 illustrates the face stimuli (the top panel) and a sample trial procedure (the bottom panel). Each trial began with a 1-s blank and then a 2-s video clip showing dynamic facial emotion from neutral to full-intensity expression. The video clip was programmed to play automatically. Participants were instructed to press the space key as soon as possible when they identified the emotion expression. The video clip stopped instantly once the participant made a keypress. An instruction for the six response keys was displayed on the screen (numeric keypad: 1 for “Happy”, 2 for “Anger”, 3 for “Sadness”, 4 for “Surprise”, 5 for “Fear”, and 6 for “Disgust”). After the participant pressed the corresponding key, the accuracy and reaction times of their response were recorded. If the video had finished but the participant had not yet responded, the screen stayed at the full intensity of the expression (the last frame). The next trial did not begin until the participant pressed the space bar and made a keypress response. Participants received one practice trial to familiarize themselves with the six response keys for the six corresponding basic emotions before the formal trial. The practice trial adopted static cartoon face images of “Happy”, “Anger”, “Sadness”, “Surprise”, “Fear”, and “Disgust” expressions of full intensity (See Figure 1).

### 2.5. Expression Imitation Task

#### 2.5.1. Apparatus and Stimuli

We ran the task using a 15.6″ laptop computer (Acer TMP259-M5726, Acer Inc.,Taipei, ROC). The iMotions^TM^ Affectiva (i-Motions, Ltd., Copenhagen, Denmark) software was utilized to record and analyze the facial imitations of participants synchronously. We selected static images of six basic emotions for one female and one male face from the Taiwan Facial Expression Image Database TFEID [56]. We created an Expression Imitation trial slide show with the facial images of the six basic emotions (happy, anger, sadness, fear, surprise, and disgust) and their corresponding verbal labels (in Chinese characters) with PowerPoint. A Chinese verbal label was first shown, followed by its corresponding facial emotion image, one after another. The Chinese characters were 1.5 cm (height) × 1.2 cm (width) in size; the face images in frontal view were 14 cm (height) × 11 cm (width) in size. The slide show was then converted into a short clip containing six expression imitation trials for each gender, which was 1 min and 40 s long. Female face trials were for female participants, while male face trials were for male participants.

#### 2.5.2. Procedures

**Figure 2:**
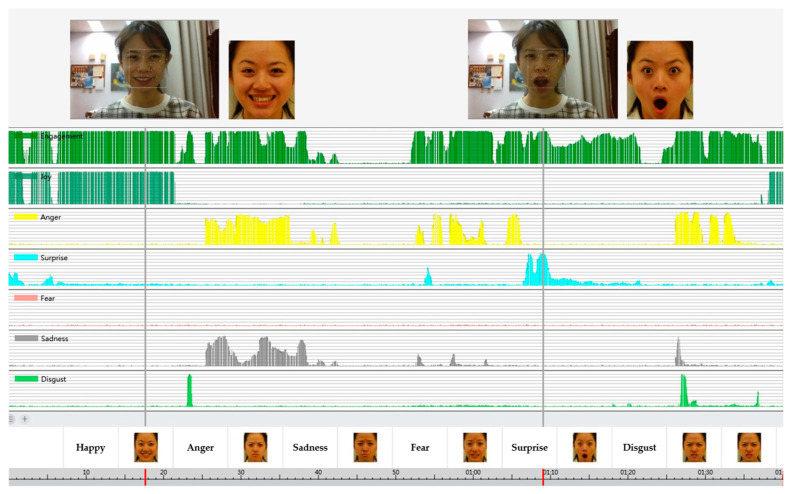
Illustration of the Expression Imitation Task. The upper part shows a participant’s facial imitation (**left**) when watching the expression imitation trial slide show (**right**); the lower part shows the interface of the iMotions^TM^ Affectiva software, presenting the real-time analysis of a participant’s facial imitation.

Each participant sat before the laptop computer and adjusted their height to fixate on the screen center. The first trial began with “Happy (開心)”, followed by the other five expressions in the order of “Anger (生氣), Sadness (傷心), Fear (害怕), Surprise (驚訝), and Disgust (噁心)”. Figure 2 illustrates the procedure and the simultaneous analysis by the iMotions^TM^ Affectiva. The trial began with a verbal label of “Happy (開心)” for 5 s, then a static “Happy” face image showed for 10 s. During the 15 s, the participants were instructed to imitate at least three times or as much as possible, then moved to the next trial. (see Ho et al. [59] for more methodological details). While the slide show video played, the built-in laptop camera recorded the facial imitations of participants simultaneously, and the iMotions^TM^ Affectiva software analyzed the imitation video in real time. The iMotions^TM^ Affectiva software is a computerized and automated facial expression analyzer. It captures raw emotions and detects the facial landmarks of the participants; their facial expressions were classified and transformed into numeric scores. The scores range from 0 (neutral) to 100 (full-intensity expression) (see Figure 2).

## 3. Results

### 3.1. Chinese AQ and PI20

The group mean AQ score was 19.5 (*SD* = 5.5), ranging from 6 to 34. The mean scores for females (*M* = 18.6, *SD* = 6.3) and males (*M* = 20.4, *SD* = 4.6) were not significantly different (*p* = 0.630). The group mean PI20 score was 49.0 (*SD* = 14.3), ranging from 23 to 80. The mean scores for females (*M* = 49.1, *SD* = 12.7) and males (*M* = 48.9, *SD* = 16.3) were also not significantly different (*p* = 0.242). Table 1 shows the group characteristics, mean total and dimensional AQ scores, and PI20 scores.

### 3.2. Dynamic Facial Emotion Recognition

**Figure 3:**
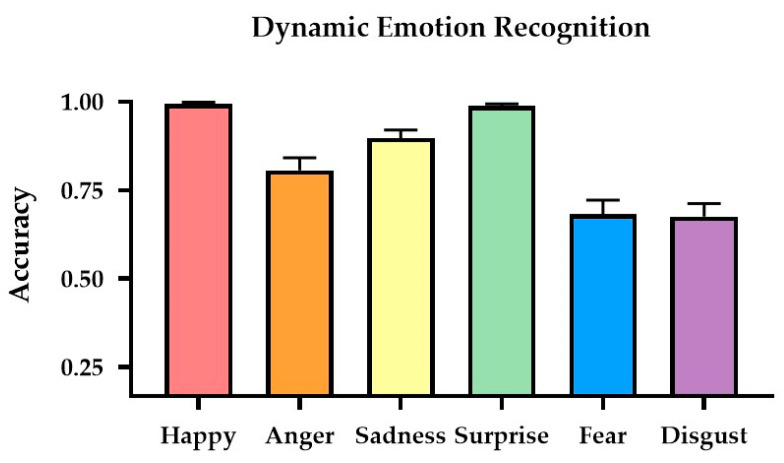
**Results of dynamic emotion recognition.** The mean recognition accuracies for the six dynamic emotion expressions.

We conducted a 2-way mixed ANOVA on the accuracy, with Gender (females, males) as the between-subject factor and Emotion Type (Happy, Anger, Sadness, Surprise, Fear, Disgust) as the within-subject factor. The main effect of Gender was not significant (*p* = 0.146); the mean accuracy for females and males was M = 0.859 (*SE* = 0.018) and M = 0.821 (*SE* = 0.018), respectively. The main effect of Emotion Type was significant (*F*(5,150) = 6.694, *p* < 0.001, *η_p_*^2^ = 0.471). From high to low, the mean accuracies for recognizing Happy, Surprise, Sadness, Anger, Fear, and Disgust were 0.994 (*SE* = 0.004), 0.988 (*SE* = 0.006), 0.897 (*SE* = 0.023), 0.806 (*SE* = 0.036), 0.681 (*SE* = 0.040), and 0.675 (*SE* = 0.037), respectively. Post hoc Scheffe tests (with an adjusted α level = 0.05/15 = 0.003) revealed that the differences between Happy and Anger (*p* < 0.001), Sadness (*p* < 0.001), Fear (*p* < 0.001), and Disgust (*p* < 0.001) were all significant. The differences between Surprise and Anger (*p* < 0.001), Sadness (*p* < 0.001), Fear (*p* < 0.001), and Disgust (*p* < 0.001) were all significant. The differences between Sadness and Fear (*p* < 0.001), and Sadness and Disgust (*p* < 0.001) were significant. The rest of the pair-wise comparisons were not significant. The Group*Emotion Type interaction effect was not significant. Figure 3 illustrates the group mean recognition accuracies for the six dynamic emotion expressions.

### 3.3. Expression Imitation

The iMotions^TM^ Affectiva software detects facial landmarks and classifies the facial expressions in return with numeric output scores for accuracy in several facial emotion categories. Here, we employed the maximum score to represent the imitation performance. The numerical scores were values between 0 (no expression) and 100 (expression fully present). The range of individuals’ raw output scores for different expressions expanded over 6 log units. Therefore, we applied logarithm transformation (log of 10). We then conducted a 2-way mixed ANOVA with Group and Emotion Type on the log Max expression scores.

**Figure 4:**
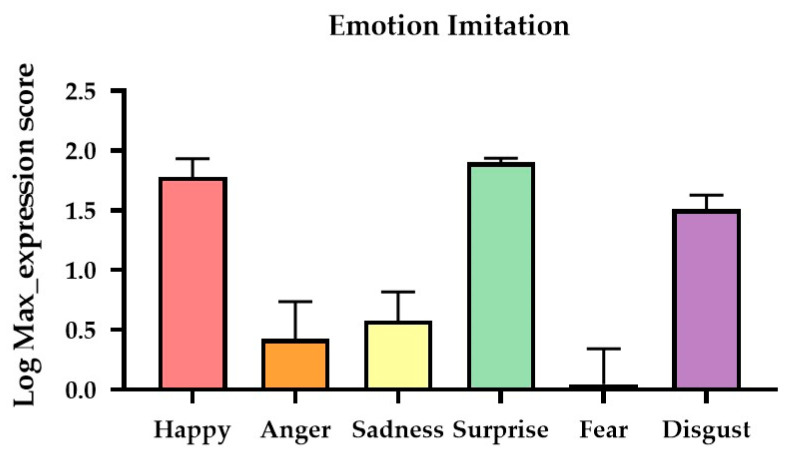
**Results of expression imitation.** The mean log Max expression score for the six emotion expressions.

The main effect of Gender was not significant (*p* = 0.40); the mean log Max expression scores for females and males were M = 1.140 (*SE* = 0.162) and M = 0.948 (*SE* = 0.157), respectively. The Emotion Type main effect was significant (*F*(5,145) = 15.436, *p* < 0.001, *η_p_*^2^ = 0.347). From high to low, the mean log Max expression scores for Surprise, Happy, Disgust, Sadness, Anger, and Fear were 1.902 (*SE* = 0.032), 1.778 (*SE* = 0.156), 1.513 (*SE* = 0.118), 0.598 (*SE* = 0.215), 0.431 (*SE* = 0.316), and 0.040 (*SE* = 0.300), respectively. Post hoc Scheffe tests (with an adjusted α level = 0.05/15 = 0.003) revealed that the differences between Happy and Anger (*p* = 0.001), Sadness (*p* < 0.001), and Fear (*p* < 0.001) were all significant. The differences between Surprise and Anger (*p* < 0.001), Sadness (*p* < 0.001), and Fear (*p* < 0.001) were all significant. The differences between Disgust and Anger (*p* = 0.002), Sadness (*p* = 0.001), and Fear (*p* < 0.001) were all significant. The rest of the pair-wise comparisons were not statistically significant. The Group*Emotion Type interaction effect was not significant. Figure 4 illustrates the group mean log Max expression scores for the six basic emotions.

### 3.4. Correlations Among AQ, PI20, and Task Performances

To examine whether individual performances on dynamic emotion recognition and expression imitation are associated with the level of autistic traits and self-rated face recognition ability, we conducted Pearson correlations to explore the associations among autistic traits (AQ total and dimensional scores), subjective rating on face recognition proficiency (PI20), performance of the dynamic facial emotion recognition (accuracy for each emotion), and performance of the expression imitation (log Max expression scores for each emotion).

#### 3.4.1. AQ, PI20, and Dynamic Emotion Recognition

**Figure 5:**
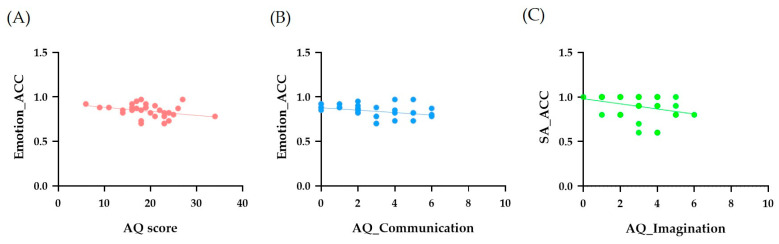
The correlations between AQ and Dynamic Emotion Recognition. Panels (**A**,**B**) show both the total AQ and the communication score negatively correlates with the mean accuracy of the dynamic emotion recognition. Panel (**C**) shows the imagination score negatively correlates with the accuracy for “sadness”.

Table 2 shows the correlations among the AQ scores (total and dimensional), PI20, and the accuracies for the dynamic emotion recognition task. We observed a significant negative correlation between the total AQ score and the mean accuracy of emotion recognition (*r* = −0.337, *p* = 0.030), indicating that the neurotypical adults with higher AQ scores tended to make more errors in dynamic emotion recognition. Among the five AQ dimensional scores (i.e., social skills, attention switching, attention to detail, communication, and imagination), the communication score was negatively correlated with emotion recognition accuracy (*r* = −0.339, *p* = 0.029). The imagination score was also negatively correlated with the accuracy for Sadness (*r* = −0.330, *p* = 0.033), meaning that adults who exhibited higher autistic traits in communication were more incapable of recognizing emotion, and those who were less imaginative showed more difficulties in recognizing Sadness (See Figure 5).

**Figure 6:**
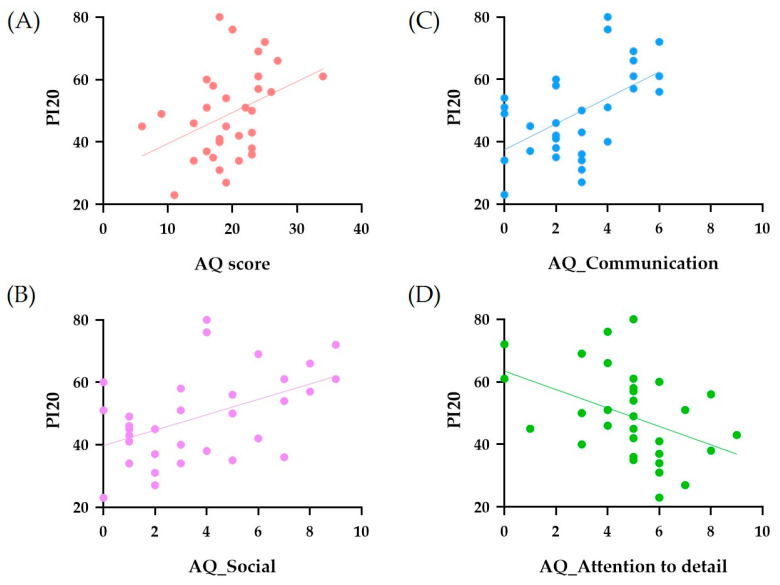
The correlations between AQ and PI20. Panels (**A**–**C**) show that the total AQ score, the social, and the communication scores positively correlate with PI20. Panel (**D**) shows the attention to detail score correlates with PI20 negatively.

In addition, there was a significant positive correlation between total AQ score and PI20 score (*r* = 0.382, *p* = 0.016), indicating that the adults with higher AQ may tend to be less proficient in face recognition by self-rating. Among the five dimensions, both social skills (*r* = 0.478, *p* = 0.003) and communication (*r* = 0.546, *p* = 0.001) scores correlated positively with the PI20 score, meaning that adults who exhibited more problems with social skills and communication were more self-conscious about difficulties in recognizing people. Interestingly, attention to detail correlated negatively with PI20 score (*r* = −0.422, *p* = 0.008), meaning that adults who tended to pay more attention to detail had better self-evaluated face recognition. None of the correlations between PI20 and the accuracies of dynamic emotion recognition reached statistical significance (See Figure 6).

#### 3.4.2. AQ, PI20, and Expression Imitation

Table 3 shows the correlations among the AQ scores (total and dimensional), PI20, and the maximum accuracies for the expression imitation task. Although the individual’s AQ total score did not correlate with the performance of the expression imitation, the dimension scores of communication (*r* = 0.336, *p* = 0.032) and imagination (*r* = 0.303, *p* = 0.049) positively correlated with the log Max expression score for imitating “surprise”, indicating that those who exhibited higher autistic trait in communication and imagination tended to make the expression of surprise very well. Furthermore, the log Max expression score for surprise correlated positively with PI20 (*r* = 0.473, *p* = 0.004), meaning that those who felt more difficulty recognizing faces were more readily able to express surprise.

#### 3.4.3. Emotion Recognition and Expression Imitation

We also examined the bivariate correlations between the performance of dynamic facial emotion recognition and facial expression imitation for each of the six basic emotions and found some interesting results (see Table 4). The log maximum score of imitation for “Anger” positively correlated with the recognition accuracies for two negative emotions, “Sadness” (*r* = 0.321, *p* = 0.039), “Disgust” (*r* = 0.375, *p* = 0.019), and the mean accuracy of six basic emotions (*r* = 0.311, *p* = 0.044) significantly. This indicated that those who were more accurate at identifying “Sadness” and “Disgust” expressions from others also tended to make more accurate “Anger” expressions.

## 4. Discussion

This study utilized the Autism-Spectrum Quotient (AQ), the Twenty Item Prosopagnosia Index (PI20), and two computerized tasks to investigate how neurotypical adults recognize dynamic emotions and imitate facial expressions. Additionally, we explored the relationships among AQ scores, PI20 scores, emotion recognition, and facial expression imitation. We obtained several noteworthy findings. For the dynamic emotion recognition, recognizing “Happy” was the easiest; on the other hand, “Anger”, “Fear”, and “Disgust” expressions were more challenging. Regarding facial expression imitation, it is easiest to make a “Surprise” expression, followed by “Happy”, while imitating “Sadness”, “Anger”, and “Fear”, which were less accurate. There was no gender difference in either emotion recognition or expression imitation performance. Finally, the correlation analysis showed that the AQ score had a positive correlation with the PI20, while it negatively correlated with the overall accuracy in recognizing the six basic emotions. This suggests that adults with higher levels of autistic traits tend to experience more difficulties with facial recognition and are less accurate in identifying dynamic emotions. The correlations among task performances and the five AQ dimension scores were also examined. Below, we will discuss these results and their implications and limitations.

Impairments in reciprocal social communication and interaction are key diagnostic features of Autism Spectrum Disorder (ASD). A deficiency in emotion processing is one reason that individuals with ASD may struggle in their daily social lives. It is proposed that autistic traits exist on a continuum within the general population. In this context, the broader autistic phenotype (BAP), which refers to subclinical characteristics, demonstrates mild but distinct features of ASD [45]. In this study, we found that an individual’s AQ score negatively correlated with the overall recognition accuracy of the six basic emotions, supporting the concept of broader autistic phenotype (BAP) that healthy adults with higher autistic traits also demonstrate similar difficulties in recognizing emotional expressions. Our observation is consistent with the findings of Poljac et al. (2013), who used dynamic morphing face stimuli to investigate the recognition of facial emotion expressions in neurotypical individuals with high or low levels of autism traits (measured by AQ) [46]. Their results demonstrated a reduced accuracy and a decreased sensitivity for the emotions of anger, disgust, and sadness in the high-AQ group, indicating that individuals with higher autistic traits generally experienced difficulties in recognizing these particular emotional expressions and that they required more intense expressions to do so, as compared to the individuals with lower autistic trait. In our study, the score of the communication subscale also negatively correlated with the overall recognition accuracy; individuals with poor communication (i.e., higher score) were less accurate in recognizing emotions (i.e., lower accuracy). Compared to other dimensions of autistic traits, communication ability might be of particular importance in differentiating emotions and is the core deficit in clinical ASD. The communication subscale reflects the ability to engage in reciprocal communication, understand conversational cues, and interpret social language nuances. Additionally, we found that the score of the imagination subscale negatively correlated with recognizing “sadness”, suggesting that less imaginative individuals (i.e., higher score) were also less accurate in identifying “sadness” expressions (i.e., lower accuracy). The imagination subscale focuses on imaginative thinking, such as the capacity for pretend play, hypothetical thinking, and enjoyment of fiction or creative scenarios. In other words, it reflects the ability to mentalize—one of the most lacking cognitive faculties in individuals with ASD. Boraston et al. tested “happiness”, “anger”, “sadness”, and “fear” recognition in adults with autism and found a deficit only in “sadness” recognition from both abstract animations and facial expressions. The impairment in the animations task significantly correlated with the degree of impairment in reciprocal social interaction, assessed by the Autism Diagnostic Observation Schedule (ADOS). This is consistent with our study that autistic traits influenced sadness recognition. The difference is that, in our research, the worse the imagination, the poorer the recognition of sadness [60].

It is important to note that the AQ score positively correlated with the PI-20 score, as we expected, meaning that a higher level of autistic trait is associated with more self-rated face recognition problems in daily encounters. Individuals with ASD have been known for their difficulties in recognizing faces. Using a within-subject design, Stantić et al. [61] applied the Cambridge Face Memory Test (CFMT), a standard test for face memory; the Glasgow Face Matching Test (GFMT), a standard test of face perception; the Oxford Face Matching Test (OFMT), a test of face perception; and 20-Item Prosopagnosia to evaluate both face memory and face perception in autistic individuals. Their results showed that face memory and face perception in autism were impaired. What is more important, individuals with ASD also self-reported more problems with face recognition on the PI-20. In our study, neurotypical individuals with higher autistic traits reported more difficulties recognizing faces. Therefore, our findings further verified the influence of autistic traits on face recognition. Developmental prosopagnosia, or congenital facial blindness, is a face recognition disorder. The 20-item prosopagnosia index (PI-20) was a self-reporting method to screen potential patients’ conditions. A Simplified Chinese Version has been published and showed good internal consistency reliability, test–retest reliability, and good validity [62]. In our study, we used our newly translated traditional Chinese Version of PI-20 from the original English Version published by Shah et al. in 2015 [54]. We observed a significant correlation between AQ and PI-20 in a moderate sample of neurotypical adults, which indicates that our translated version has good validity.

In this study, we used the built-in engagement index of iMotions^TM^ Affectiva software as an approximation for expression imitation, that is, by detecting dozens of landmarks on muscles of facial expression to categorize the types of expressions and to quantify the participant’s level of imitation. In the present study, the AQ scores did not appear to influence the overall performance in expression imitation. However, the ability to imitate “surprise” expression was positively correlated with two AQ subscales, “communication”, “imagination”, and PI-20. This study found that “happiness” had the highest recognition accuracy among the six basic emotions, which aligns with findings from previous studies. However, in the expression imitation task, both “surprise” and “happiness” demonstrated the highest imitation accuracy, while the other four expressions scored relatively low. According to Russell’s circumplex model of affect, which is based on two fundamental neurophysiological systems, valence (a pleasure–displeasure continuum) and arousal (or alertness), “surprise” and “happiness” were more clustered together than the other four negative emotions [63]. In addition to the six basic emotions, compound emotions, which were formed by combining two or more basic emotions (e.g., happily surprised, sadly fearful, or angrily disgusted), could be produced and distinguished. To achieve a higher detection rate, both the AU codes and the configural (second-order) features must be discriminative [64]. One reason we observed relatively poor imitation accuracy for sadness, anger, and fear could be the limitation of the iMotions^TM^ Affectiva. The iMotions^TM^ Affectiva is trained to recognize emotions from posed facial expression databases and mostly from Caucasians. However, facial emotion recognition can be susceptible to challenges in real-world situations, such as different races, occlusion, head orientations, and illumination. A more recent automatic FER model was developed to overcome these challenges and be more applicable in real-life scenarios [65]. The accuracy of the iMotions Affectiva may decrease when being applied to detect natural, spontaneous expressions of emotions of various races [66]; these software limitations might lead to the relatively lower accuracy for the above-mentioned expressions in the present results.

Notably, we found that individuals who score high with AQ “communication” and “imagination” are also good at mimicking “surprise” expressions. How can we explain this phenomenon? “Surprise” has been understood as a pre-affective state or an emotion that can be neutral or mildly negative [67]. According to the cognitive–evolutionary model, unexpectedness or novelty often evokes “surprise”. The “surprising” events interrupt the ongoing progress and lead to a shift in attention [68]. Additionally, through a metacognitive estimate, any factor that increases difficulties explaining an abnormal event results in a higher level of “surprise” [69]. Hence, it is reasonable to infer that people who struggle with communication, are less imaginative, or have difficulties recognizing faces might often feel interrupted and consume more energy to explain when encountering unexpected events. This insight suggests that improving communication and imagination skills could lead to better perception and a deeper understanding of emotional cues.

We originally expected that individual levels of expression imitation would positively correlate with the performance of dynamic emotion recognition. Many studies revealed the importance of emotion imitation. According to Lipps’ emotion recognition model based on the facial feedback hypothesis, facial mimicry affects the observer’s emotional state. Individuals exposed to emotional facial expressions tend to report emotional states congruent with these displays [70]. According to Kowallik et al., imitation is a positive within-participant predictor of recognition accuracy in the imitation block, and emotion recognition improvements were larger in people with higher levels of autistic traits [49]. The heterogeneity of participants in this study might not be large enough to show such a discrepancy. A recent meta-analysis study also demonstrated that stronger facial mimicry responses were positively related to a higher level of empathy but not facial emotion recognition [71]. In this study, we only found significant correlations between imitating “Anger” and recognizing “Sadness”, “Disgust”, and the overall accuracy of the six basic emotions, suggesting that individuals who were more accurate at identifying “Sadness” and “Disgust” tended to show stronger facial muscle activities (log Max engagement) when imitating “Anger”. Many studies have reported that it was easy to confuse “Anger” with “Disgust” when asked to judge posed facial expressions. According to FACS by Ekman et al., the “facial expression of disgust” with a so-called “nose scrunch” consisted of either or both Action Unit (AU) 9 (wrinkling of the upper nose) and 10 (raising of the upper lip and wrinkling of the lower nose) [72]. When tested by the face with the “nose scrunch”, most observers chose “disgust” followed by “angry” [73]. Further explorations revealed that observers tended to judge the “nose scrunch” as expressing “Disgust” when the preceding set included an anger scowl, but as “Anger” when the anger scowl was omitted. When the anger scowl was omitted, and a sick face was included, a greater proportion of observers judged the nose scrunch as “Anger” [74]. “Sadness” and “Anger” are both thought to play an integral role in depression [75]. The underlying link between these three negative emotions needs further exploration.

## 5. Conclusions, Limitations, and Future Work

In conclusion, the present study was among the first to explore AQ traits, emotion recognition, and expression imitation in neurotypical adults using commercial software (iMotions^TM^ Affectiva) based on the facial action unit coding system. Among the six basic emotions, “happiness” was the easiest to recognize; “anger”, “fear”, and “disgust” expressions were more challenging. Making the expressions of “surprise” and “happiness” showed the highest accuracy, followed by “disgust”; the accuracy of imitating “sadness”, “anger”, and “fear” was much lower. With a moderate sample size, we observed that the AQ score positively correlated with the PI-20 and negatively correlated with the overall recognition accuracy of the six basic emotions, indicating that adults with higher autistic traits tended to report more problems with faces and were less accurate in recognizing dynamic emotions. The present study was subject to some limitations, such as the small sample size, the homogeneity in the neurotypical group, the bias associated with the self-reporting method, and the fact that we used the built-in reading of the iMotions^TM^ Affectiva as an index of expression imitation rather than using electromyography (EMG) to detect muscle movements directly. Due to recent evidence showing equivalent effectiveness of both measures [76], we are confident that the absence of EMG recordings shall not undermine our results.

Nevertheless, we plan to explore a newer facial emotion recognition (FER) model developed for robot vision in the future, which excels at analyzing participants’ natural expression imitation [65]. We will also recruit individuals with ASD to better understand how autistic traits influence emotion processing, including recognition and production. By establishing the similarities and differences between autistic and non-autistic groups, we aim to highlight a potential intervention for enhancing social interaction capacity in individuals with clinical and sub-clinical ASD.

## Figures and Tables

**Table 1:** The group mean scores of the Chinese Autism Quotient and PI20 (with standard deviations in parentheses).

	Females (n = 16)	Males (n = 16)	All (n = 32)
Age (years)	22.6 (3.7)	23.2 (2.7)	22.9 (3.2)
AQ score	18.6 (6.3)	20.4 (4.6)	19.5 (5.5)
Social skill	3.3 (3.0)	4.3 (2.6)	3.8 (2.8)
Attention switching	5.3 (1.8)	5.1 (2.0)	5.2 (1.9)
Attention to detail	5.3 (1.5)	4.6 (2.5)	4.9 (2.1)
Communication	2.3 (2.0)	3.3 (1.7)	2.8 (1.9)
Imagination	2.6 (1.5)	3.3 (1.5)	2.9 (1.5)
PI20 score	49.1 (12.7)	48.9 (16.3)	49.0 (14.3)

**Table 2:** The correlations among the AQ scores (total and dimensional), PI20, and the dynamic emotion recognition task accuracies.

	Autism-Spectrum Quotient (AQ)						Dynamic Facial Emotion Recognition Task							PI20
	AQ Score 1	Social Skill	Attention Switching	Attention to Detail	Communication	Imagination	HA_ ACC ^2^	AN_ ACC ^3^	SA_ ACC ^4^	SU_ ACC ^5^	FE_ ACC ^6^	DI_ ACC ^7^	Emotion_ACC	
**Social skill**	0.763 (0.000) **	1	0.254 (0.080)	−0.411 (0.010) **	0.680 (0.000) **	0.336 (0.030) *	0.118 (0.260)	0.032 (0.431)	−0.171 (0.175)	−0.035 (0.426)	−0.183 (0.157)	−0.050 (0.393)	−0.144 (0.216)	0.478 (0.003) **
**Attention switching**	0.633 (0.000) **	-	1	−0.062 (0.368)	0.484 (0.003) **	0.072 (0.347)	−0.113 (0.270)	−0.149 (0.207)	−0.219 (0.114)	−0.013 (0.473)	0.067 (0.357)	−0.183 (0.158)	−0.193 (0.145)	0.212 (0.122)
**Attention to detail**	−0.001 (0.497)	-	-	1	−0.306 (0.044) *	−0.148 (0.209)	−0.012 (0.474)	−0.014 (0.469)	0.263 (0.073)	−0.064 (0.363)	−0.038 (0.418)	−0.163 (0.186)	−0.031 (0.433)	−0.422 (0.008) **
**Communication**	0.786 (0.000) **	-	-	-	1	0.178 (0.165)	0.039 (0.416)	−0.048 (0.397)	−0.292 (0.053)	−0.096 (0.301)	−0.255 (0.079)	−0.219 (0.114)	−0.339 (0.029) *	0.546 (0.001) **
**Imagination**	0.471 (0.003) **	-	-	-	-	1	−0.011 (0.476)	−0.140 (0.223)	−0.330 (0.033) *	−0.208 (0.127)	−0.041 (0.412)	−0.128 (0.242)	−0.253 (0.081)	0.157 (0.195)
**HA_ACC** ^2^	0.025 (0.445)	-	-	-	-	-	1	−0.256 (0.078)	0.094 (0.304)	−0.098 (0.298)	−0.192 (0.146)	0.094 (0.305)	−0.086 (0.320)	0.091 (0.309)
**AN_ACC ^3^**	−0.095 (0.303)	-	-	-	-	-	-	1	−0.124 (0.250)	−0.133 (0.234)	−0.195 (0.143)	0.128 (0.242)	0.343 (0.027) *	−0.220 (0.113)
**SA_ACC ^4^**	−0.257 (0.078)	-	-	-	-	-	-	-	1	0.505 (0.002) **	0.084 (0.325)	0.221 (0.112)	0.432 (0.007) **	−0.074 (0.344)
**SU_ACC ^5^**	−0.137 (0.227)	-	-	-	-	-	-	-	-	1	0.135 (0.230)	0.092 (0.309)	0.269 (0.068)	−0.040 (0.414)
**FE_ACC ^6^**	−0.179 (0.163)	-	-	-	-	-	-	-	-	-	1	0.397 (0.012) *	0.631 (0.000) **	−0.029 (0.437)
**DI_ACC ^7^**	−0.262 (0.074)	-	-	-	-	-	-	-	-	-	-	1	0.811 (0.000) **	0.003 (0.493)
**Emotion_ACC**	−0.337 (0.030) *	-	-	-	-	-	-	-	-	-	-	-	1	−0.120 (0.256)
**PI20**	0.382 (0.016) *	-	-	-	-	-	-	-	-	-	-	-	-	1

Significant difference: *, *p* < 0.05; **, *p* < 0.01. 1 AQ score of Chinese Autism-Spectrum Quotient (adult version). ^2−7^ Performance indices of dynamic facial emotion recognition: ^2^ HA_ACC: Accuracy for HAPPY. ^3^ AN_ACC: Accuracy for ANGER. ^4^ SA_ACC: Accuracy for SADNESS. ^5^ SU_ACC: Accuracy for SURPRISE. ^6^ FE_ACC: Accuracy for FEAR. ^7^ DI_ACC: Accuracy for DISGUST.

**Table 3:** The correlations among the AQ scores (total and dimensional), PI20, and the maximum accuracies for the expression imitation task.

	Autism-Spectrum Quotient (AQ)						Facial Emotion Imitation Task							PI20
	AQ Score 1	Social Skill	Attention Switching	Attention to Detail	Communication	Imagination	HA_Max ^2^	AN_Max ^3^	SA_Max ^4^	SU_Max ^5^	FE_Max ^6^	DI_Max ^7^	Emotion_Max	
**Social skill**	0.763 (0.000) **	1	0.254 (0.080)	−0.411 (0.010) **	0.680 (0.000) **	0.336 (0.030) *	0.260 (0.079)	0.118 (0.264)	0.066 (0.361)	0.098 (0.299)	−0.320 (0.040) *	−0.265 (0.075)	−0.045 (0.405)	0.478 (0.003) **
**Attention switching**	0.633 (0.000) **	-	1	−0.062 (0.368)	0.484 (0.003) **	0.072 (0.347)	0.237 (0.100)	−0.035 (0.427)	−0.246 (0.091)	0.133 (0.238)	−0.189 (0.154)	−0.106 (0.286)	−0.144 (0.219)	0.212 (0.122)
**Attention to detail**	−0.001 (0.497)	-	-	1	−0.306 (0.044) *	−0.148 (0.209)	−0.001 (0.497)	−0.144 (0.219)	−0.239 (0.097)	−0.209 (0.129)	−0.047 (0.402)	−0.012 (0.475)	−0.185 (0.160)	−0.422 (0.008) **
**Communication**	0.786 (0.000) **	-	-	-	1	0.178 (0.165)	0.228 (0.109)	0.011 (0.477)	−0.047 (0.401)	0.336 (0.032)*	−0.150 (0.211)	−0.087 (0.321)	−0.025 (0.448)	0.546 (0.001) **
**Imagination**	0.471 (0.003) **	-	-	-	-	1	−0.101 (0.294)	0.187 (0.156)	0.033 (0.430)	0.303 (0.049) *	0.134 (0.236)	0.183 (0.162)	0.181 (0.165)	0.157 (0.195)
**HA_Max** ^2^	0.258 (0.081)	-	-	-	-	-	1	−0.237 (0.099)	0.100 (0.296)	−0.143 (0.222)	0.088 (0.318)	−0.051 (0.392)	0.177 (0.170)	0.162 (0.192)
**AN_Max ^3^**	0.042 (0.411)	-	-	-	-	-	-	1	0.114 (0.271)	0.305 (0.048) *	0.416 (0.010) **	0.151 (0.208)	0.675 (0.000) **	0.121 (0.258)
**SA_Max ^4^**	−0.150 (0.211)	-	-	-	-	-	-	-	1	0.132 (0.240)	0.267 (0.074)	−0.003 (0.494)	0.554 (0.001) **	0.255 (0.083)
**SU_Max ^5^**	0.208 (0.131)	-	-	-	-	-	-	-	-	1	0.329 (0.035) *	0.029 (0.439)	0.354 (0.025) *	0.473 (0.004) **
**FE_Max ^6^**	−0.257 (0.081)	-	-	-	-	-	-	-	-	-	1	0.357 (0.024) *	0.827 (0.000) **	−0.030 (0.436)
**DI_Max ^7^**	−0.159 (0.197)	-	-	-	-	-	-	-	-	-	-	1	0.390 (0.015) *	−0.189 (0.154)
**Emotion_Max**	−0.105 (0.286)	-	-	-	-	-	-	-	-	-	-	-	1	0.160 (0.195)
**PI20**	0.382 (0.016) *	-	-	-	-	-	-	-	-	-	-	-	-	1

Significant difference: *, *p* < 0.05; **, *p* < 0.01. 1 AQ score of Chinese Autism-Spectrum Quotient (adult version). ^2−7^ Performance indices for facial emotion imitation: ^2^ HA_Max: Log Max_expression score for HAPPY. ^3^ AN_Max: Log Max_expression score for ANGER. ^4^ SA_Max: Log Max_expression score for SADNESS. ^5^ SU_Max: Log Max_expression score for SURPRISE. ^6^ FE_Max: Log Max_expression score for FEAR. ^7^ DI_Max: Log Max_expression score for DISGUST.

**Table 4:** The bi-variate correlations between the performance of dynamic facial emotion recognition and facial expression imitation for each of the six basic emotions.

	Dynamic Facial Emotion Recognition Task							Facial Emotion Imitation Task						
	HA_ACC 1	AN_ACC ^2^	SA_ACC ^3^	SU_ACC ^4^	FE_ACC ^5^	DI_ACC ^6^	Emotion_ACC	HA_Max ^7^	AN_Max ^8^	SA_Max ^9^	SU_Max ^10^	FE_Max ^11^	DI_Max ^12^	Emotion_Max
**HA_ACC** 1	1	−0.256 (0.078)	0.094 (0.304)	−0.098 (0.298)	−0.192 (0.146)	0.094 (0.305)	−0.086 (0.320)	−0.068 (0.357)	0.126 (0.250)	−0.013 (0.472)	−0.134 (0.236)	0.033 (0.430)	−0.116 (0.267)	0.026 (0.444)
**AN_ACC ^2^**	-	1	−0.124 (0.250)	−0.133 (0.234)	−0.195 (0.143)	0.128 (0.242)	0.343 (0.027) *	0.209 (0.130)	0.070 (0.354)	−0.022 (0.453)	−0.136 (0.233)	0.137 (0.231)	0.164 (0.189)	0.155 (0.203)
**SA_ACC ^3^**	-	-	1	0.505 (0.002) **	0.084 (0.325)	0.221 (0.112)	0.432 (0.007) **	−0.115 (0.270)	0.321 (0.039) *	−0.033 (0.430)	−0.138 (0.229)	−0.124 (0.253)	−0.180 (0.166)	0.018 (0.461)
**SU_ACC ^4^**	-	-	-	1	0.135 (0.230)	0.092 (0.309)	0.269 (0.068)	0.236 (0.100)	−0.018 (0.461)	−0.121 (0.258)	−0.194 (0.148)	−0.132 (0.240)	0.015 (0.469)	−0.062 (0.369)
**FE_ACC ^5^**	-	-	-	-	1	0.397 (0.012) *	0.631 (0.000) **	−0.074 (0.347)	−0.012 (0.475)	0.235 (0.102)	0.029 (0.439)	0.054 (0.386)	−0.098 (0.301)	0.069 (0.356)
**DI_ACC ^6^**	-	-	-	-	-	1	0.811 (0.000) **	0.009 (0.480)	0.375 (0.019) *	0.050 (0.394)	−0.104 (0.289)	0.094 (0.308)	−0.030 (0.436)	0.226 (0.111)
**Emotion_ACC**	-	-	-	-	-	-	1	0.033 (0.431)	0.311 (0.044) *	0.125 (0.252)	−0.144 (0.220)	0.085 (0.324)	−0.045 (0.405)	0.219 (0.118)
**HA_Max ^7^**	-	-	-	-	-	-	-	1	−0.237 (0.099)	0.100 (0.296)	−0.143 (0.222)	0.088 (0.318)	−0.051 (0.392)	0.177 (0.170)
**AN_Max ^8^**	-	-	-	-	-	-	-	-	1	0.114 (0.271)	0.305 (0.048) *	0.416 (0.010) **	0.151 (0.208)	0.675 (0.000) **
**SA_Max ^9^**	-	-	-	-	-	-	-	-	-	1	0.132 (0.240)	0.267 (0.074)	−0.003 (0.494)	0.554 (0.001) **
**SU_Max ^10^**	-	-	-	-	-	-	-	-	-	-	1	0.329 (0.035) *	0.029 (0.439)	0.354 (0.025) *
**FE_Max ^11^**	-	-	-	-	-	-	-	-	-	-	-	1	0.357 (0.024) *	0.827 (0.000) **
**DI_Max ^12^**	-	-	-	-	-	-	-	-	-	-	-	-	1	0.390 (0.015) *
**Emotion_Max**	-	-	-	-	-	-	-	-	-	-	-	-	-	1

Significant difference: *, *p* < 0.05; **, *p* < 0.01. ^1−6^ Performance indices of dynamic facial emotion recognition: 1 HA_ACC: Accuracy for HAPPY. ^2^ AN_ACC: Accuracy for ANGER. ^3^ SA_ACC: Accuracy for SADNESS. ^4^ SU_ACC: Accuracy for SURPRISE. ^5^ FE_ACC: Accuracy for FEAR. ^6^ DI_ACC: Accuracy for DISGUST. ^7−12^ Performance indices for facial emotion imitation: ^7^ HA_Max: Log Max_expression score for HAPPY. ^8^ AN_Max: Log Max_expression score for ANGER. ^9^ SA_Max: Log Max_expression score for SADNESS. ^10^ SU_Max: Log Max_expression score for SURPRISE. ^11^ FE_Max: Log Max_expression score for FEAR. ^12^ DI_Max: Log Max_expression score for DISGUST.

## Data Availability

The original datasets (as an Excel file) were analyzed during the study, and the main figures and tables have been publicly available and stored at Open Science Framework via https://osf.io/9nt5a/.

## References

[1] Edition F. (2013). Diagnostic and statistical manual of mental disorders. Am. Psychiatr. Assoc..

[2] Kanner L. (1943). Autistic disturbances of affective contact. Nerv. Child.

[3] Behrmann M., Thomas C., Humphreys K. (2006). Seeing it differently: Visual processing in autism. Trends Cogn. Sci..

[4] Tanaka J.W., Sung A. (2016). The “eye avoidance” hypothesis of autism face processing. J. Autism Dev. Disord..

[5] Chien S.H.-L., Wang L.-H., Chen C.-C., Chen T.-Y., Chen H.-S. (2014). Autistic children do not exhibit an own-race advantage as compared to typically developing children. Res. Autism Spectr. Disord..

[6] Dawson G., Carver L., Meltzoff A.N., Panagiotides H., McPartland J., Webb S.J. (2002). Neural correlates of face and object recognition in young children with autism spectrum disorder, developmental delay, and typical development. Child Dev..

[7] Dawson G., Webb S.J., McPartland J. (2005). Understanding the nature of face processing impairment in autism: Insights from behavioral and electrophysiological studies. Dev. Neuropsychol..

[8] Gross T.F. (2004). The perception of four basic emotions in human and nonhuman faces by children with autism and other developmental disabilities. J. Abnorm. Child Psychol..

[9] Buitelaar J.K., Van der Wees M., Swaab–Barneveld H., Van der Gaag R.J. (1999). Theory of mind and emotion-recognition functioning in autistic spectrum disorders and in psychiatric control and normal children. Dev. Psychopathol..

[10] Crespi B., Badcock C. (2008). Psychosis and autism as diametrical disorders of the social brain. Behav. Brain Sci..

[11] Frith U. (2003). Autism: Explaining the Enigma.

[12] Loveland K.A., Tunali-Kotoski B., Pearson D.A., Brelsford K.A., Ortegon J., Chen R. (1994). Imitation and expression of facial affect in autism. Dev. Psychopathol..

[13] Drimalla H., Baskow I., Behnia B., Roepke S., Dziobek I. (2021). Imitation and recognition of facial emotions in autism: A computer vision approach. Mol. Autism.

[14] Oberman L.M., Winkielman P., Ramachandran V.S. (2009). Slow echo: Facial EMG evidence for the delay of spontaneous, but not voluntary, emotional mimicry in children with autism spectrum disorders. Dev. Sci..

[15] McIntosh D.N., Reichmann-Decker A., Winkielman P., Wilbarger J.L. (2006). When the social mirror breaks: Deficits in automatic, but not voluntary, mimicry of emotional facial expressions in autism. Dev. Sci..

[16] Yoshimura S., Sato W., Uono S., Toichi M. (2015). Impaired overt facial mimicry in response to dynamic facial expressions in high-functioning autism spectrum disorders. J. Autism Dev. Disord..

[17] Weiss E.M., Rominger C., Hofer E., Fink A., Papousek I. (2019). Less differentiated facial responses to naturalistic films of another person’s emotional expressions in adolescents and adults with High-Functioning Autism Spectrum Disorder. Prog. Neuro-Psychopharmacol. Biol. Psychiatry.

[18] Morton J., Johnson M.H. (1991). CONSPEC and CONLERN: A two-process theory of infant face recognition. Psychol. Rev..

[19] Chien S.H.-L. (2011). No more top-heavy bias: Infants and adults prefer upright faces but not top-heavy geometric or face-like patterns. J. Vis..

[20] Chien S.H.-L., Hsu H.Y. (2012). No more top-heavy bias: On early specialization process for face and race in infants. Chin. J. Clin. Psychol..

[21] Barrera M.E., Maurer D. (1981). The perception of facial expressions by the three-month-old. Child Dev..

[22] Walker-Andrews A.S. (1997). Infants’ perception of expressive behaviors: Differentiation of multimodal information. Psychol. Bull..

[23] Denham S.A., Gullotta T.P., Bloom M., Kotch J., Blakely C., Bond L., Adams G., Browne C., Klein W., Ramos J. (2003). Social and Emotional Learning, Early Childhood. Encyclopedia of Primary Prevention and Health Promotion.

[24] Herba C., Phillips M. (2004). Annotation: Development of facial expression recognition from childhood to adolescence: Behavioural and neurological perspectives. J. Child Psychol. Psychiatry.

[25] Herba C.M., Landau S., Russell T., Ecker C., Phillips M.L. (2006). The development of emotion-processing in children: Effects of age, emotion, and intensity. J. Child Psychol. Psychiatry.

[26] Richoz A.-R., Lao J., Pascalis O., Caldara R. (2018). Tracking the recognition of static and dynamic facial expressions of emotion across the life span. J. Vis..

[27] Kolb B., Wilson B., Taylor L. (1992). Developmental changes in the recognition and comprehension of facial expression: Implications for frontal lobe function. Brain Cogn..

[28] Ekman P., Friesen W.V. (1971). Constants across cultures in the face and emotion. J. Pers. Soc. Psychol..

[29] Izard C.E. (2001). Emotional intelligence or adaptive emotions?. Emotion.

[30] Lawrence K., Campbell R., Skuse D. (2015). Age, gender, and puberty influence the development of facial emotion recognition. Front. Psychol..

[31] Chiang Y.-C., Chien S.H.-L., Lyu J.-L., Chang C.-K. (2024). Recognition of dynamic emotional expressions in children and adults and its associations with empathy. Sensors.

[32] Thomas L.A., De Bellis M.D., Graham R., LaBar K.S. (2007). Development of emotional facial recognition in late childhood and adolescence. Dev. Sci..

[33] Riddell C., Nikolić M., Dusseldorp E., Kret M.E. (2024). Age-related changes in emotion recognition across childhood: A meta-analytic review. Psychol. Bull..

[34] Meltzoff A.N., Moore M.K. (1977). Imitation of facial and manual gestures by human neonates. Science.

[35] Meltzoff A.N., Moore M.K. (1983). Newborn infants imitate adult facial gestures. Child Dev..

[36] Meltzoff A.N., Marshall P.J. (2018). Human infant imitation as a social survival circuit. Curr. Opin. Behav. Sci..

[37] Field T.M., Walden T.A. (1982). Production and discrimination of facial expressions by preschool children. Child Dev..

[38] Grossard C., Chaby L., Hun S., Pellerin H., Bourgeois J., Dapogny A., Ding H., Serret S., Foulon P., Chetouani M. (2018). Children facial expression production: Influence of age, gender, emotion subtype, elicitation condition and culture. Front. Psychol..

[39] Dimberg U. (1982). Facial reactions to facial expressions. Psychophysiology.

[40] Blairy S., Herrera P., Hess U. (1999). Mimicry and the judgment of emotional facial expressions. J. Nonverbal Behav..

[41] Wallbott H.G. (1991). Recognition of emotion from facial expression via imitation? Some indirect evidence for an old theory. Br. J. Soc. Psychol..

[42] Oberman L.M., Winkielman P., Ramachandran V.S. (2007). Face to face: Blocking facial mimicry can selectively impair recognition of emotional expressions. Soc. Neurosci..

[43] Rychlowska M., Cañadas E., Wood A., Krumhuber E.G., Fischer A., Niedenthal P.M. (2014). Blocking mimicry makes true and false smiles look the same. PLoS ONE.

[44] Wood A., Rychlowska M., Korb S., Niedenthal P. (2016). Fashioning the face: Sensorimotor simulation contributes to facial expression recognition. Trends Cogn. Sci..

[45] Piven J., Palmer P., Jacobi D., Childress D., Arndt S. (1997). Broader autism phenotype: Evidence from a family history study of multiple-incidence autism families. Am. J. Psychiatry.

[46] Poljac E., Poljac E., Wagemans J. (2013). Reduced accuracy and sensitivity in the perception of emotional facial expressions in individuals with high autism spectrum traits. Autism.

[47] Fujimori M., Okanoya K. (2013). An ERP study of autistic traits and emotional recognition in non-clinical adolescence. Psychology.

[48] Kerr-Gaffney J., Mason L., Jones E., Hayward H., Ahmad J., Harrison A., Loth E., Murphy D., Tchanturia K. (2020). Emotion recognition abilities in adults with anorexia nervosa are associated with autistic traits. J. Clin. Med..

[49] Kowallik A.E., Pohl M., Schweinberger S.R. (2021). Facial imitation improves emotion recognition in adults with different levels of sub-clinical autistic traits. J. Intell..

[50] Faul F., Erdfelder E., Buchner A., Lang A.-G. (2009). Statistical power analyses using G* Power 3.1: Tests for correlation and regression analyses. Behav. Res. Methods.

[51] Ethical Principles of Psychologists and Code of Conduct American Psychological Association. https://www.apa.org/ethics/code/.

[52] Liu M. (2008). Screening adults for asperger syndrome and high-functioning autism by using the Autism-Spectrum Quotient (AQ)(Mandarin Version). J. Spec. Educ..

[53] Baron-Cohen S., Wheelwright S., Skinner R., Martin J., Clubley E. (2001). The autism-spectrum quotient (AQ): Evidence from asperger syndrome/high-functioning autism, malesand females, scientists and mathematicians. J. Autism Dev. Disord..

[54] Shah P., Gaule A., Sowden S., Bird G., Cook R. (2015). The 20-item prosopagnosia index (PI20): A self-report instrument for identifying developmental prosopagnosia. R. Soc. Open Sci..

[55] Tsantani M., Vestner T., Cook R. (2021). The Twenty Item Prosopagnosia Index (PI20) provides meaningful evidence of face recognition impairment. R. Soc. Open Sci..

[56] Taiwanese Facial Expression Image Database Institute of Brain Science, National Yang-Ming University. Brain Mapping Laboratory, Taipei. https://bmlab.web.nycu.edu.tw/%e8%b3%87%e6%ba%90%e9%80%a3%e7%b5%90/.

[57] Chien S.H.-L., Tai C.-L., Yang S.-F. (2018). The development of the own-race advantage in school-age children: A morphing face paradigm. PLoS ONE.

[58] Chen C., Yang S., Chien S. (2016). Exploring the own-race face encoding advantage and the other-race face categorization bias in Taiwanese adults: Using a morphing face paradigm. Chin. J. Clin. Psychol..

[59] Ho M.W.-R., Chien S.H.-L., Lu M.-K., Chen J.-C., Aoh Y., Chen C.-M., Lane H.-Y., Tsai C.-H. (2020). Impairments in face discrimination and emotion recognition are related to aging and cognitive dysfunctions in Parkinson’s disease with dementia. Sci. Rep..

[60] Boraston Z., Blakemore S.-J., Chilvers R., Skuse D. (2007). Impaired sadness recognition is linked to social interaction deficit in autism. Neuropsychologia.

[61] Stantić M., Ichijo E., Catmur C., Bird G. (2022). Face memory and face perception in autism. Autism.

[62] Sun W., Wang Y., Wang J., Luo F. Psychometric Properties of the Chinese version of the 20-item Prosopagnosia Index (PI20). Proceedings of the E3S Web of Conferences.

[63] Russell J.A. (1980). A circumplex model of affect. J. Pers. Soc. Psychol..

[64] Du S., Tao Y., Martinez A.M. (2014). Compound facial expressions of emotion. Proc. Natl. Acad. Sci. USA.

[65] Liu H., Zhou Q., Zhang C., Zhu J., Liu T., Zhang Z., Li Y.-F. (2024). MMATrans: Muscle movement aware representation learning for facial expression recognition via transformers. IEEE Trans. Ind. Inform..

[66] Dupré D., Andelic N., Morrison G., McKeown G. Accuracy of three commercial automatic emotion recognition systems across different individuals and their facial expressions. Proceedings of the 2018 IEEE International Conference on Pervasive Computing and Communications Workshops (PerCom Workshops).

[67] Noordewier M.K., Breugelmans S.M. (2013). On the valence of surprise. Cogn. Emot..

[68] Reisenzein R., Horstmann G., Schützwohl A. (2019). The cognitive-evolutionary model of surprise: A review of the evidence. Top. Cogn. Sci..

[69] Foster M.I., Keane M.T. (2015). Why some surprises are more surprising than others: Surprise as a metacognitive sense of explanatory difficulty. Cogn. Psychol..

[70] Lipps T. (1905). Das wissen von fremden Ichen. Psychologische Untersuchungen.

[71] Holland A.C., O’Connell G., Dziobek I. (2021). Facial mimicry, empathy, and emotion recognition: A meta-analysis of correlations. Cogn. Emot..

[72] Ekman P., Friesen W.V. (1978). Facial action coding system. Environ. Psychol. Nonverbal. Behav..

[73] Russell J.A. (1994). Is there universal recognition of emotion from facial expression? A review of the cross-cultural studies. Psychol. Bull..

[74] Pochedly J.T., Widen S.C., Russell J.A. (2012). What emotion does the “facial expression of disgust” express?. Emotion.

[75] Bourke C., Douglas K., Porter R. (2010). Processing of facial emotion expression in major depression: A review. Aust. N. Z. J..

[76] Kulke L., Feyerabend D., Schacht A. (2020). A comparison of the Affectiva iMotions Facial Expression Analysis Software with EMG for identifying facial expressions of emotion. Front. Psychol..

